# Volunteer trials of a novel improvised dry decontamination protocol for use during mass casualty incidents as part of the UK’S Initial Operational Response (IOR)

**DOI:** 10.1371/journal.pone.0179309

**Published:** 2017-06-16

**Authors:** Richard Amlôt, Holly Carter, Lorna Riddle, Joanne Larner, Robert P. Chilcott

**Affiliations:** 1 Emergency Response Department Science & Technology, Public Health England, Porton Down, Salisbury, Wiltshire, United Kingdom; 2 Research Centre for Topical Drug Delivery and Toxicology, School of Pharmacy, University of Hertfordshire, Hatfield, United Kingdom; EPA, UNITED STATES

## Abstract

Previous studies have demonstrated that rapid evacuation, disrobing and emergency decontamination can enhance the ability of emergency services and acute hospitals to effectively manage chemically-contaminated casualties. The purpose of this human volunteer study was to further optimise such an “Initial Operational Response” by (1) identifying an appropriate method for performing improvised skin decontamination and (2) providing guidance for use by first responders and casualties. The study was performed using two readily available, absorbent materials (paper towels and incontinence pads). The decontamination effectiveness of the test materials was measured by quantifying the amount of a chemical warfare agent simulant (methyl salicylate) removed from each volunteer’s forearm skin. Results from the first study demonstrated that simulant recovery was lower in all of the dry decontamination conditions when compared to matched controls, suggesting that dry decontamination serves to reduce chemical exposure. Blotting in combination with rubbing was the most effective form of decontamination. There was no difference in effectiveness between the two absorbent materials. In the following study, volunteers performed improvised dry decontamination, either with or without draft guidelines. Volunteers who received the guidance were able to carry out improvised dry decontamination more effectively, using more of the absorbent product (blue roll) to ensure that all areas of the body were decontaminated and avoiding cross-contamination of other body areas by working systematically from the head downwards. Collectively, these two studies suggest that absorbent products that are available on ambulances and in acute healthcare settings may have generic applicability for improvised dry decontamination. Wherever possible, emergency responders and healthcare workers should guide casualties through decontamination steps; in the absence of explicit guidance and instructions, improvised dry decontamination may not be performed correctly or safely.

## Introduction

The UK has a well-established capability for responding to mass casualty incidents that involve the release of noxious contaminants. Traditionally, this capability has involved a “one size fits all” approach using wet decontamination to remove contaminants from the skin, in specialised decontamination showering units that can be deployed at the scene of an incident [1]. A significant limitation of this approach is that the deployment of mass decontamination units requires a team of specialist responders, and can take several hours under some circumstances. Since some chemicals may be lethal within minutes [2], it is necessary to develop rapid decontamination procedures that can be put in place prior to the arrival of specialist teams and equipment and that can be initiated by the first (non-specialist) emergency responders to arrive at the scene.

Recent research has identified means of improving existing methods for reducing the potential health effects of exposure to such dangerous substances [3]. In particular, research funded by the UK Department of Health has identified that improvised or interim decontamination options exist that could minimise injury and/or illness if initiated within the first 15–20 min from exposure. Such steps include early removal of contaminated clothing, and dry or wet decontamination using available absorbent materials or water sources. For some time, emergency services have been able to perform so-called “rinse-wipe-rinse” methods of improvised wet decontamination; however, until recently, dry decontamination has primarily been limited to military use, with fuller’s earth being the most commonly utilised dry decontamination product. Thus, dry decontamination is a relatively new intervention in civilian emergency response settings, and there is limited evidence available regarding the best products or methods for dry decontamination of affected casualties. There have also been few opportunities to examine the public acceptability of dry decontamination methods, a factor which may affect adherence to decontamination protocols. The success of emergency decontamination has been shown to be associated with emergency responders communicating health-focused, practical information about the need for decontamination [4–6]. Poor communication strategies may result in non-compliance with responders and increased risk from primary and secondary contamination due to poorly conducted disrobing and decontamination protocols.

The current research project has sought to identify the most effective product for improvised dry decontamination in civilian settings, such as on-scene emergency response and acute healthcare facilities. The previous laboratory phase of this project identified absorbent materials that are readily available within the National Health Service (NHS)—e.g. tissue paper (or “blue roll”), gauze dressings and incontinence pads—and that could be used to remove a contaminant from the skin. The five most promising products identified were then evaluated against a range of toxic industrial chemicals and simulants in order to quantify their relative effectiveness as decontaminants using an established in vitro test system [7]. From this laboratory research, the two best performing products were chosen to take forward to the human volunteer trials described in this report.

### Study aims and objectives

The main objective was to confirm the most effective method for improvised dry decontamination using materials widely available in ambulance and hospital settings. Two human volunteer trials were conducted to address this objective. Firstly, *Study 1* assessed the most efficacious method of use (blotting, rubbing, or blotting and rubbing) for the two products identified (blue roll and incontinence pad). Two groups of participants performed each of the different methods, one using blue roll and the other incontinence pads. Decontamination effectiveness was measured in terms of the removal of a simulant contaminant (methyl salicylate) applied to the forearms of volunteers. Outcomes from *Study 1* informed the development of a guidance document for emergency responders on the management of dry decontamination, the effectiveness of which was tested during *Study 2*.

*Study 2* had two aims. The new draft dry decontamination guidance was tested by asking two groups of participants to perform dry decontamination, with one group receiving the guidance and the other group receiving no guidance. Participants were observed carrying out the dry decontamination process under both conditions, and adherence to the decontamination protocol was assessed and compared between the two groups. Secondly, public perceptions of the acceptability of dry decontamination and participants’ willingness to comply with the decontamination process were assessed. It was expected that those in the guidance group would report higher perceptions of acceptability of using dry decontamination, as well as greater willingness to comply with responder instructions during a real incident. Taken together, the outcomes of *Study 1* and *Study 2* could provide evidence to support the adoption of an improvised dry decontamination protocol using proprietary absorbent materials readily available in NHS settings, and guide emergency responders on the most effective casualty management strategy for these novel decontamination approaches.

## Methodology

### Study 1

#### Study design

This study was independently approved by the NHS South Central–Hampshire A Research Ethics Committee. The study used a 2 × 3 mixed factorial design. The between-subjects factor was product type with two levels: blue roll and incontinence pad. The within-subjects factor was decontamination method, which had three levels: blotting, rubbing, and blotting and rubbing. Twenty participants carried out each of the three different decontamination methods, using either blue roll or incontinence pads.

#### Participants

A total of 20 volunteers took part in the study, 11 males (55%) and 9 females (45%), all aged over 18. Prior to inclusion in the study, each volunteer received a medical screening form, designed to exclude those individuals with pre-existing health concerns that could affect their participation in the study. Participants received £30 in high street gift vouchers as a reward for their participation.

#### Materials

**Fig 1:**
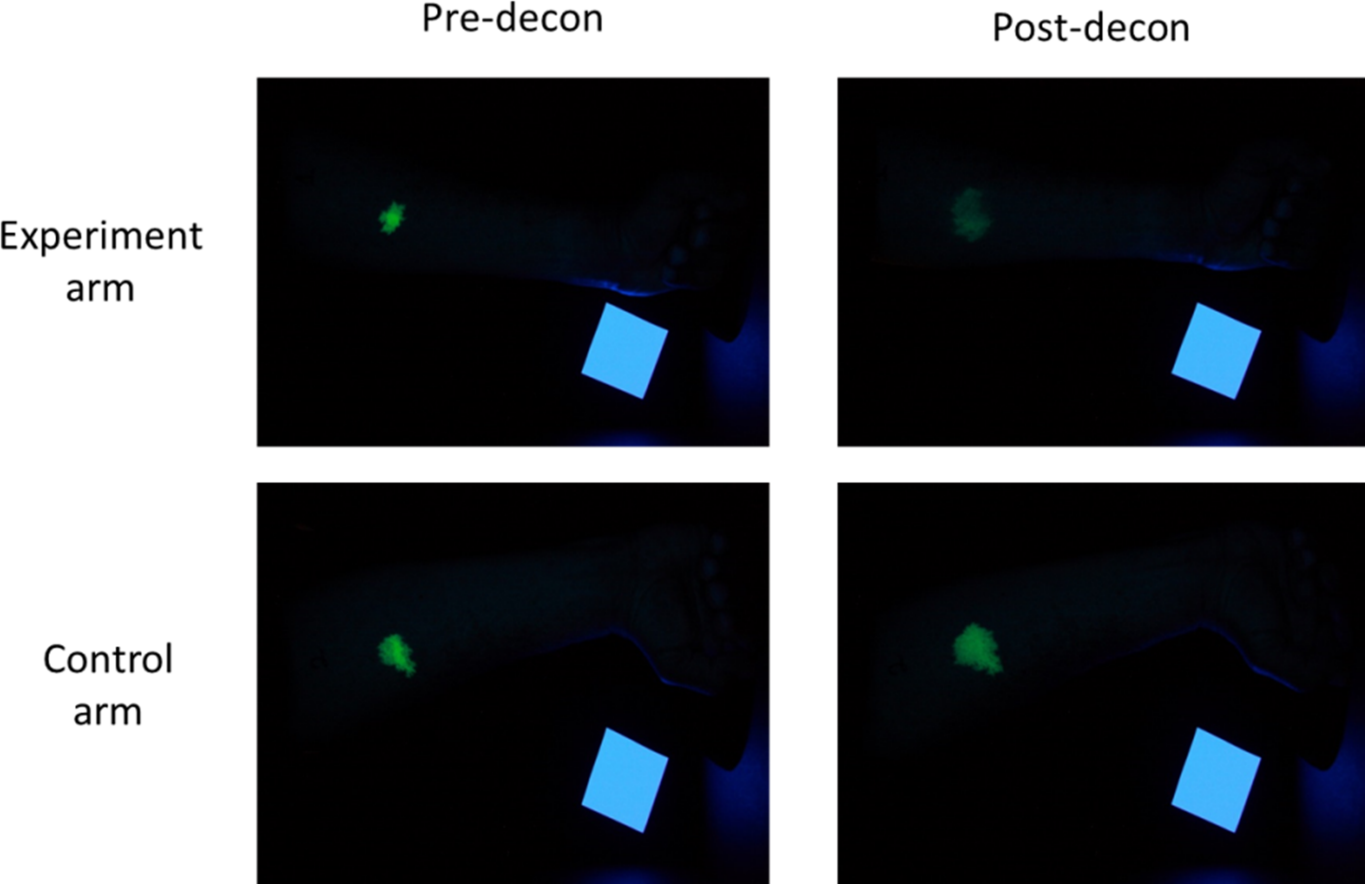
Illustrative pre- and post-decontamination UV image for experimental arm and control arm for one participant in *Study 1*. A white reference square (25 cm^2^) can be seen in each image.

The simulant contaminant was a solution of 10 mg of curcumin per 1 mL of 99.9% methyl salicylate (Fisher Scientific, UK). This concentration was based on a series of pilot tests designed to identify the optimum solution of the two substances in combination that would allow effective recovery of the simulant from the skin of volunteers, and visualisation of the simulant using UV-illuminated photography. This solution has been used successfully in a previous study of emergency decontamination [8]. Methyl salicylate has a long history of use in human volunteer studies and is used as a simulant for the chemical warfare agent sulphur mustard [9]. Curcumin fluoresces under ultra violet (UV) illumination when applied in conjunction with a methyl salicylate solvent. Ten microlitres of methyl salicylate was applied to each participant’s fore-arms in each study session (20 μL total per session). Participants were asked to place their fore-arm through a hole near the bottom of a lightproof box containing 100 UV light bulbs and four UV filter squares. A camera fitted to the top of the box then took photographs of the fluorescent simulant so that the spread of the simulant could be measured (see Fig 1).

The blue roll used in this study was 1-ply blue roll of the type commonly found on ambulances and in healthcare settings. The blue roll was cut into squares of 10 × 10 cm, and then folded twice to create a 4-ply 25 cm^2^ square. The incontinence pad used in this study was of a type used by the ambulance service (MoliNea® Plus Underpad; Paul Hartmann Ltd., Heywood, UK). The incontinence pads were cut into squares of 25 cm^2^.

#### Procedure

**Fig 2:**
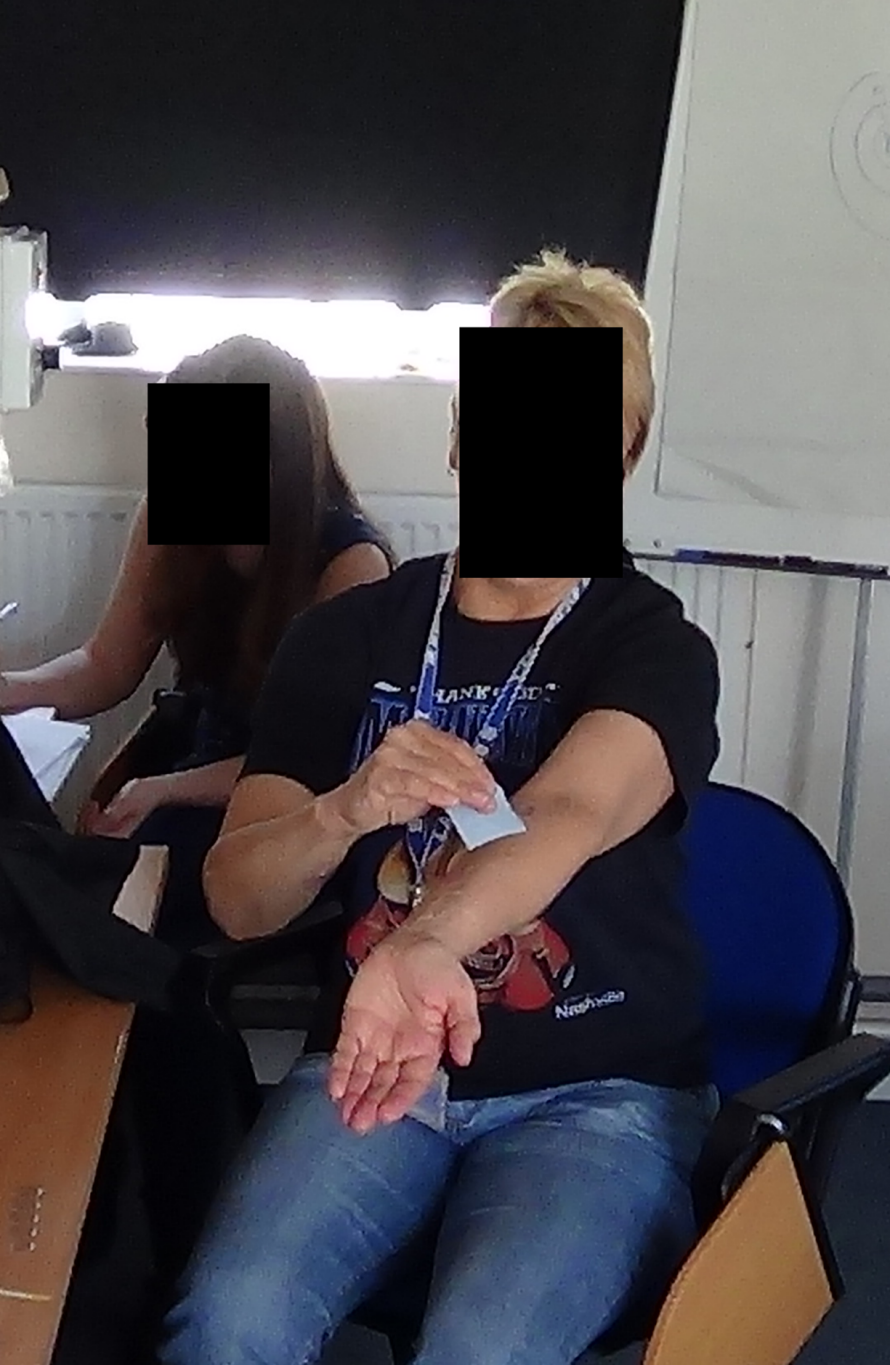
Participant carrying out dry decontamination using blue roll during *Study 1*.

All twenty participants took part in three separate study sessions over the course of three weeks. In each session, volunteers arrived at the study site, were briefed on their involvement in the study, and signed a consent form. Participants were asked to wear a short-sleeved or sleeveless top during the study. Initially, participants were asked to place their arms into the UV box, and a baseline fluorescent image was taken. A 25 cm^2^ square was marked on each participant’s forearms using a marker. The simulant was applied to the centre of both marked locations using a pipette. Each participant’s dominant arm served as the control site, allowing them to use this arm to carry out dry decontamination on the opposite forearm. Immediately following simulant application, participants were asked to place their arms into the UV box, and a second photo was taken. After 13 minutes, participants were again asked to place their arms into the UV box, to monitor the spread of the simulant immediately prior to decontamination. At 15 minutes following simulant application, participants were provided with one of the two dry decontamination products (blue roll or incontinence pad), which were allocated randomly. Participants were asked to either just blot, just rub, or both blot and rub the simulant application site for 5 seconds, attempting to remove the simulant from their arm (see Fig 2). Each participant carried out all three of the different methods over the course of three study sessions; the order was randomised for each participant. A final UV-illuminated image was captured following decontamination.

After the final photo, the two application sites were swabbed with cotton wool soaked in ethanol. Each site was swabbed sequentially with a dry cotton wool bud, a cotton wool bud soaked in absolute ethanol, and another dry cotton wool bud. The three cotton buds per site were transferred to the same glass vial. Absolute ethanol (10 mL) was added to each vial. The same two sites were then subjected to tape-strip sampling. Five 22 mm D-Squame adhesive discs (Cuderm Corporation, USA) were applied to each site to remove sequential upper layers of the stratum corneum. The adhesive discs were applied using forceps. Uniform pressure was applied to each disc using a D-Squame applicator (Cuderm Corporation, USA). Forceps were used to remove the disc and place it into a glass vial. Absolute ethanol (5 mL) was added to each vial. Sub-sampling took place at least 24 hours from the time that ethanol was applied to the samples. Combination samples (5 mL) were prepared by mixing 1 mL aliquots from each site’s five adhesive disc samples. Aliquots (1.5 mL) were sub-sampled from each cotton-swab vial and combination adhesive-disc vial before dispatch to a collaborating laboratory for analysis.

On each study day, standard dilutions of simulant (5 μL), along with the corresponding volume of absolute ethanol described in the preceding paragraph, were applied to triplicate vials of each sample type. Blank samples were produced by applying ethanol to each type of sample. At least 24 hours later, 1.5 mL was sub-sampled from each blank sample. One standard sample was selected for dilution and sub-sampling. All standard and blank aliquots were dispatched to a collaborating laboratory, along with aliquots of samples collected on the study day. At the end of each study session participants were given the opportunity to ask any questions, before being reminded of the date, time and location of their next session. At the end of the third session of the trial, participants were debriefed and given further information about the study to take away.

#### Data analysis

For image analysis, all raw image files were converted to JPEG files using appropriate software (Digital Photo Professional v3.4.1.1, Canon) and subsequently analysed using publicly available image analysis software (ImageJ, National Institutes of Health, USA). A spatial scale was established for each individual participant by setting the known area of a 25 cm^2^ paper square placed on the participant’s arm in the first image of each session and determining the pixel count of this area. Each JPEG image was split into three 8-bit greyscale images containing the red, blue and green components of the original, the blue and red images were discarded and only the green channel was used for the analysis. The lower and upper thresholds were set at 20–255 after preliminary tests showed these to be the most appropriate values to allow the software to detect all visible fluorescence. The area of interest was selected and the fluorescence within this area was analysed (pixel size: 150-infinity, circularity: 0.00–1.00). The total area of fluorescence was reported (cm^2^) for each image. The area of simulant spread was measured for: arm 1 (experiment arm) pre-decontamination, arm 1 post-decontamination, arm 2 (control arm) prior to decontamination of arm 1, and arm 2 after decontamination of arm 1. The pre-decontamination measure was subtracted from the post-decontamination measure for each arm, to give a measure of the spread of the contaminant as a result of decontamination, for each arm. As the simulant reacted differently on each participant’s skin, it was necessary to adjust the arm 1 measure to take into account the amount of spread of the simulant on the control arm (arm 2). This was achieved by subtracting the measure of spread for arm 2 from the measure of spread for arm 1. This approach resulted in a measure of how much the simulant had spread on arm 1 (experimental) compared to arm 2 (control), and therefore measured how much the simulant had spread as a result of decontamination. A positive value indicated that decontamination resulted in increased spread of the simulant, whilst a negative value indicated that decontamination resulted in reduced spread of the contaminant.

Extracts of the solution from each of the vials containing matrices (swabs and strips) were analysed by headspace analysis using GC-MS [10], and their respective concentrations were determined. Descriptive statistics were derived for each dry decontamination method and product, before inferential statistical analysis was conducted using SPSS 22.0. Mixed two-factor analysis of variance was used to assess any differences or interactions between the product type and decontamination method, followed by planned comparisons of each decontamination method and the matched control conditions.

### Study 2

#### Design

*Study 2* used a between-subjects design. Twenty-one participants conducted a whole-body dry decontamination process, with 10 participants receiving guidance on how to complete the process (developed based on the results from *Study 1*), whereas the other 11 participants received no guidance on how to complete the process. The aim of the study was to assess the utility of the draft guidance and to explore the performance of participants conducting dry decontamination without instructions.

#### Participants

A total of 21 participants took part in *Study 2*, 16 males (76%) and 5 females (24%). All participants were aged over 18. Participants received £20 in high street gift vouchers as a reward for taking part.

#### Materials

**Fig 3:**
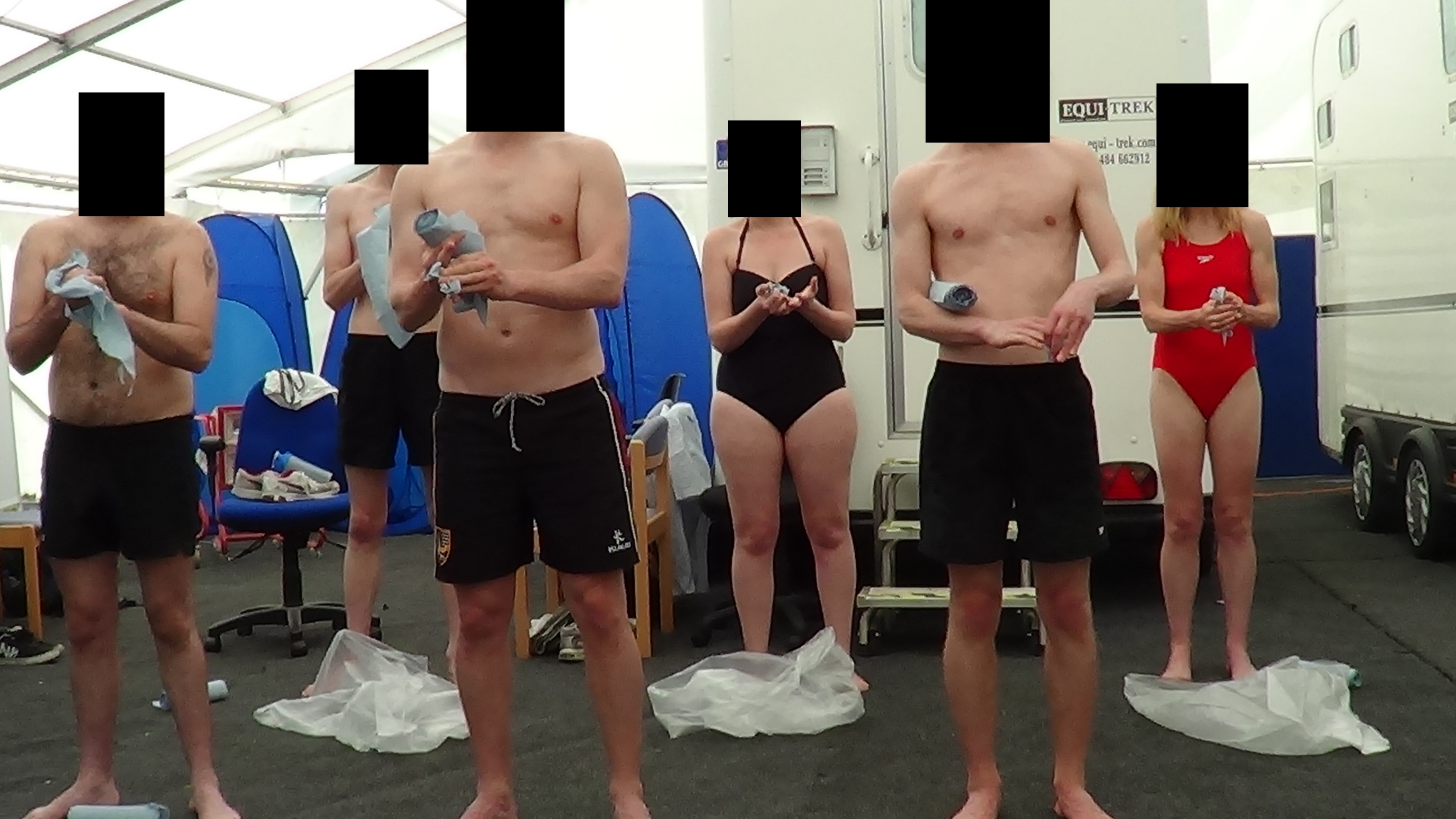
Participants carrying out dry decontamination with blue roll whilst following guidance during *Study 2*.

Participants conducted dry decontamination using blue roll. Each participant was provided with thirty sheets. Participants in the guidance group were instructed to use a separate piece of blue roll for their hands, their face and neck, their left arm, their right arm, their torso and back, their left leg and foot, and their right leg and foot. A two-page guide on the dry decontamination process was developed, based on the outcomes from *Study 1*. The guidance contained information about the nature of dry decontamination, details of when dry decontamination is necessary, and instructions for carrying out dry decontamination. The guide was used to instruct participants through the dry decontamination process in the guidance group (Fig 3). The participants in the no-guidance group were asked to clean themselves using the blue roll. S1 File contains the draft dry decontamination guidance document.

A questionnaire was developed relating to effectiveness of communication, perceived acceptability of dry decontamination as an intervention, willingness to comply with dry decontamination during a real incident, and intention to seek further treatment. The questions relating to effectiveness of communication were adapted from scales used in exercises and field trials involving wet decontamination [4, 5]. The communication scale contained four items, relating to the perceived effectiveness of explanations about dry decontamination (e.g. “I understood why I was being asked to undergo dry decontamination”) and perceived effectiveness of instructions given (e.g. “I was clear about what I was supposed to do during dry decontamination”). The scale had good internal reliability (Cronbach’s Alpha = 0.83).

Following a literature review of factors affecting the perceived acceptability of health interventions, four factors that contribute to the perceived acceptability of health interventions were identified. These were comfort during the health intervention [11,12], the intervention being quick to undertake [13], the intervention being easy to undertake [13,14], and the intervention being perceived as effective [12]. From these four identified factors, five questionnaire items designed to measure perceived acceptability were developed, as follows: two items measured how comfortable participants felt using the dry decontamination product (e.g. “I felt comfortable using the blue roll to remove the simulated contaminant from my skin”); one item measured ability to quickly perform dry decontamination (“I was able to quickly remove the simulated contaminant from my skin using the blue roll”); one item measured how easy participants found it to perform dry decontamination (“I found it easy to use the blue roll to remove the simulated contaminant from my skin”); and one item measured the perceived efficacy of dry decontamination (“I think that using blue roll is an effective way to remove the simulated contaminant from my skin”). The scale had good internal reliability (Cronbach’s Alpha = 0.72). One item measured whether participants would be confident that they were clean after carrying out dry decontamination (“If this were a real incident, I would feel confident that I was clean after using the blue roll to remove the contaminant from my skin”). One item measured whether participants would feel the need to seek further treatment after carrying out dry decontamination (“If this were a real incident, I would feel the need to seek further treatment after using the blue roll to remove the contaminant from my skin”). Another item measured whether participants would be willing to comply with the need for dry decontamination during a real incident (“I would be willing to undergo dry decontamination during a real life incident of this kind”). This item was adapted from previous questionnaires used in field trials and exercises involving wet decontamination [4,5]. The full questionnaire is included in S2 File.

#### Procedure

Volunteers arrived at the study site, were briefed on their involvement in the study, and signed a consent form. Ten participants made up the guidance group and 11 the no-guidance group. The groups’ responses were evaluated in two separate study sessions. In the guidance group, participants received information about what the product was, why it was necessary for them to use it, and detailed instructions on exactly how the product should be used; in the no-guidance group, participants were simply told to use the product to remove the “contaminant” from their skin. Participants were asked to listen to a short scenario describing an incident in which dry decontamination would be required (see S3 File for a copy of the scenario). Following the scenario, participants were sprayed with water, to simulate a contaminant. Participants carried out dry decontamination as instructed by a researcher, using the product provided. Participants were asked to place any used pieces of blue roll into a plastic bag, and researchers then counted the number of pieces of blue roll used within each group. Two video cameras were used to record participants carrying out the dry decontamination process. The trial was deemed to have finished once the last participant within each group had finished carrying out dry decontamination and had completed the post-decontamination questionnaire.

#### Data analysis

*Study 2* measures focused on examining whether participants completed the decontamination process successfully (using observational analysis) and how participants experienced the process (questionnaire). The video data from each of the two groups was subjected to observational analysis. An initial coding scheme was developed to establish behaviours of interest (e.g. that sufficient blue roll was used to avoid cross-contamination), and videos of each of the two sessions were coded to establish the proportion of each type of behaviour within each group. The results from the questionnaire data were analysed using SPSS 21.0. Items designed to measure perceived acceptability were subjected to principal components analysis to establish their suitability for use as one scale. This analysis revealed the presence of two factors with eigenvalues exceeding 1, explaining 49% and 20% of the variance. An inspection of the scree plot revealed a clear break after the first component. Further, all five items loaded more strongly onto the first factor than the second factor. The decision was therefore taken to retain only one factor, supporting the use of all five items as one measure of perceived acceptability. Differences between the two groups were tested using independent samples t-tests.

## Results

### Study 1

#### Skin surface spreading of simulant

A mixed analysis of variance was conducted to assess the impact of the two different decontamination products (blue roll and incontinence pad) and the three different methods of dry decontamination used (blotting, rubbing, and blotting and rubbing) on the skin surface spreading of the simulant contaminant. Table 1 shows the means and standard deviations of the spread of simulant for each of the three methods and for both the dry decontamination products. There was no significant interaction between product and method (F (2, 9) = 0.650, p > 0.05). The main effect for method approached significance (F (2, 9) = 3.88, p = 0.06), with a trend towards rubbing alone increasing the spread of the simulant for both blue roll and incontinence pad. There was no significant difference between the blue roll and the incontinence pad as regards the spread of the simulant (F (1, 10) = 4.36, p = 0.14).

**Table 1:** Spread of simulant for blue roll and incontinence pads, using three different methods of dry decontamination.

Decontamination method	Blue roll (cm^2^)			Incontinence pad (cm^2^)		
	*n*	*M*	*SD*	*n*	*M*	*SD*
**Blotting**	5	-4.64	9.03	7	3.85	11.98
**Rubbing**	5	4.23	4.08	7	7.77	6.20
**Blotting & Rubbing**	5	2.17	1.39	7	2.06	4.89

#### Skin swabs

**Fig 4:**
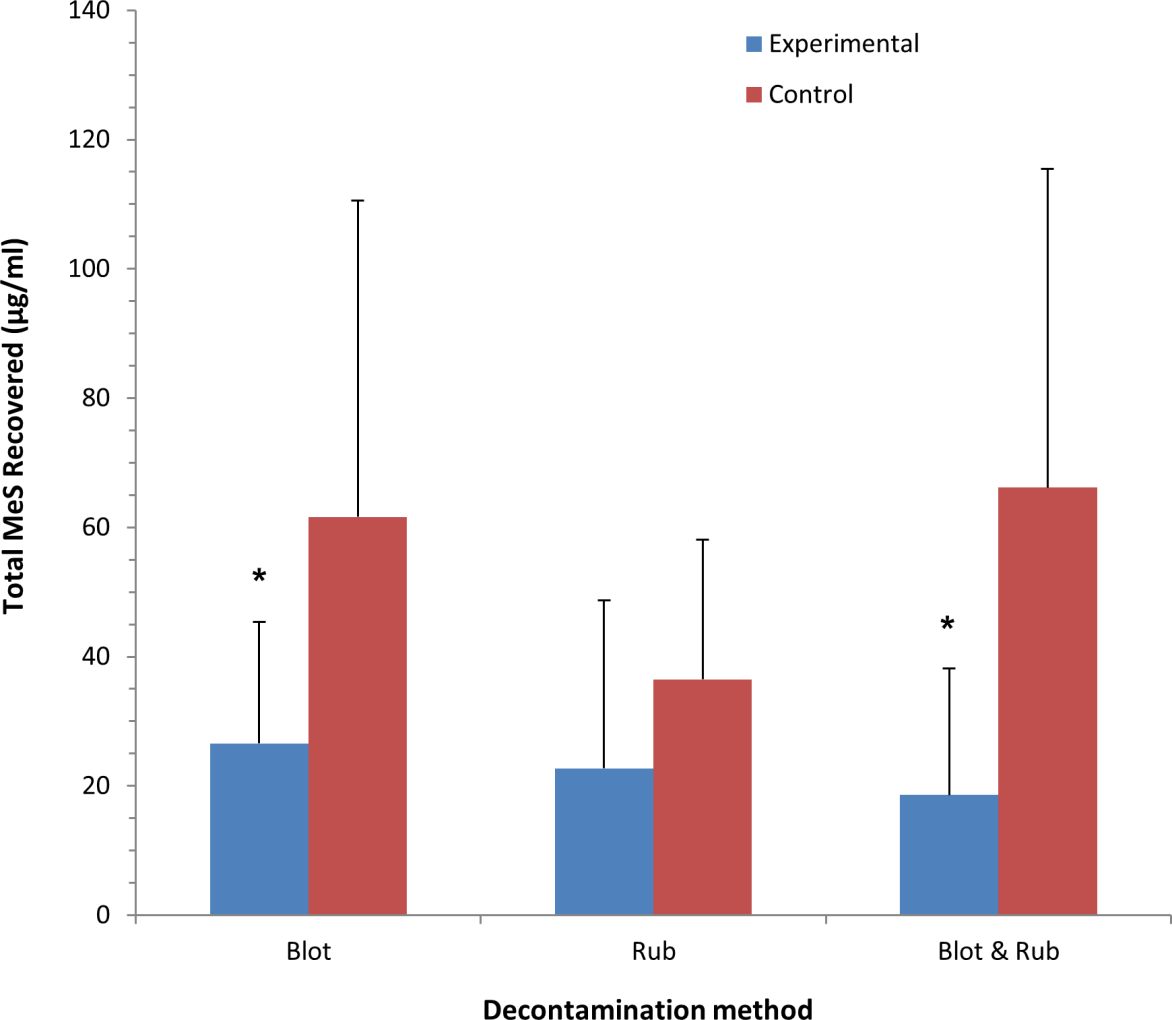
Amount of methyl salicylate recovered from forearm sites with cotton swabs by decontamination method (blotting, rubbing, and blotting and rubbing). All values are mean ± standard deviation. Asterisks indicate significant differences between experimental and control groups (*p < 0.05).

A mixed analysis of variance was conducted to assess the effectiveness of the two different dry decontamination products (blue roll and incontinence pad) and the three different dry decontamination methods used (blotting, rubbing, and blotting and rubbing) on the amount of simulant recovered in skin swabs. There was no significant interaction between product and method (F (2, 14) = 1.24, p > 0.05). The main effects for both method and product were also not significant (F (2, 14) = 0.79, p > 0.05, and F (1, 15) = 0.42, p > 0.05, respectively). As there were no significant differences between the two product types, the data for blue roll and incontinence pads were combined for subsequent analysis. Planned comparisons between experimental conditions and matched controls using paired-samples t-tests revealed that the blotting and rubbing method resulted in recovery of a significantly lower quantity of methyl salicylate from the skin surface when compared to the matched control (t(18) = -2.39, p < 0.05). The blotting method also showed a significant difference from control (t(18) = -3.09, p < 0.05). However, there was no significant difference between the rubbing method and the control condition (t(15) = -1.60, p > 0.05). The outcomes for the skin swab data are presented in Fig 4.

#### Permeation of simulant into stratum corneum

**Fig 5:**
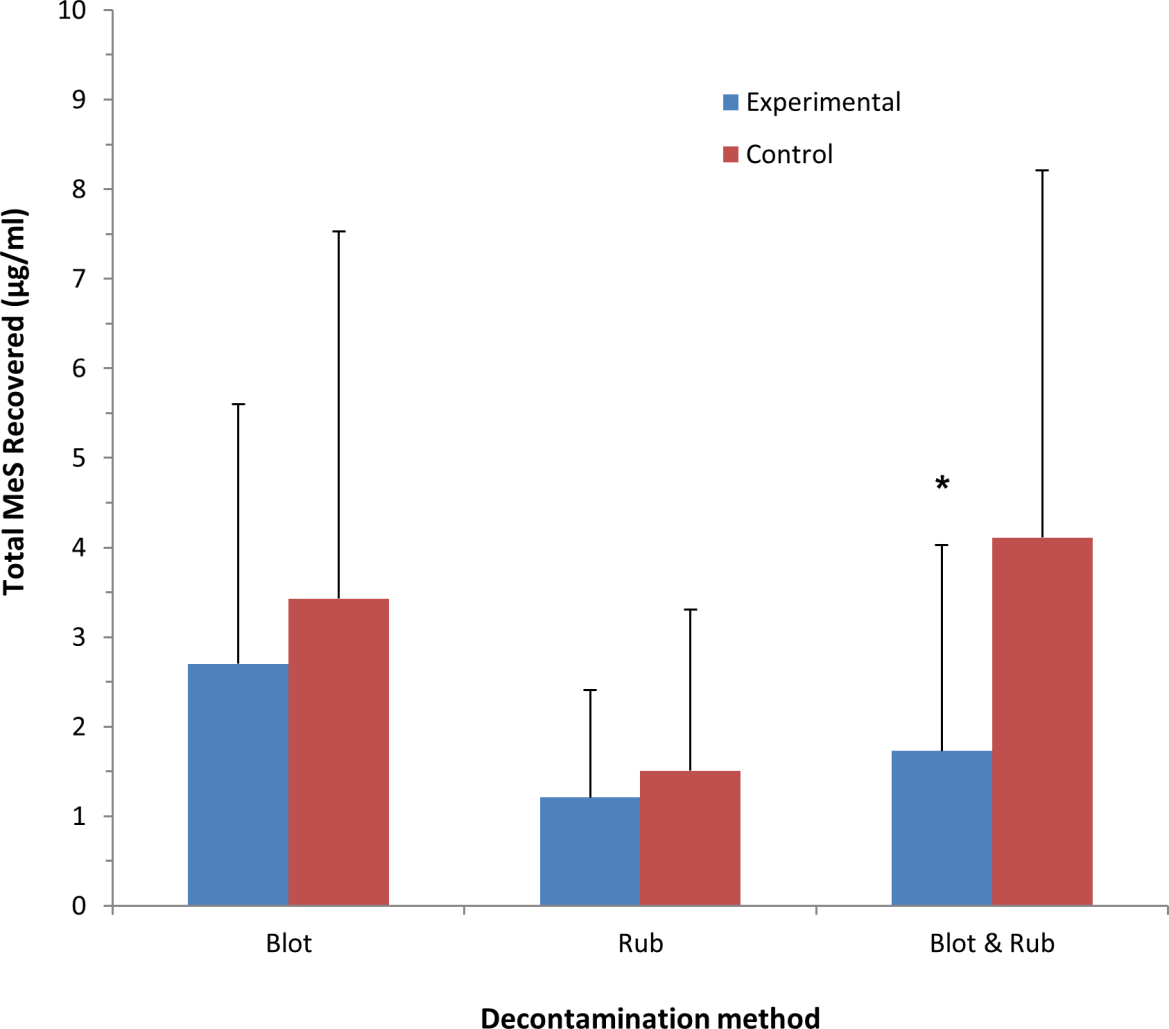
Amount of methyl salicylate recovered from forearm sites with skin strips by decontamination method (blotting, rubbing, and blotting and rubbing). All values are mean ± standard deviation. Asterisks indicate significant differences between experimental and control conditions (*p < 0.05).

There was no significant interaction between product type and decontamination method (F (2, 14) = 0.582, p > 0.05). The main effect for the decontamination method approached significance (F (2, 14) = 3.32, p = 0.07); however, the decontamination product showed no main effect (F (1, 15) = 0.16, p > 0.05). As there was no significant difference between the two product types, data for blue roll and incontinence pads were combined for subsequent analysis. Planned comparisons of the effectiveness of each dry decontamination method compared to the matched control condition revealed that the blotting and rubbing method resulted in a significantly lower amount of methyl salicylate recovered in the skin strips when compared to matched controls (t(18) = -2.39, p < 0.05). By contrast, there was no significant difference between either the blotting or rubbing only conditions and controls (p > 0.05 for both). The outcomes for the skin strip data are presented in Fig 5.

### Study 2

Several key behaviours, which would be important to ensure that the dry decontamination process was effective, were identified prior to the study. These behaviours included: ensuring that all parts of the body were decontaminated; decontaminating from the top down; and using sufficient blue roll to ensure there would be no spread of the contaminant (e.g. not using the same piece of blue roll to decontaminate more than one part of the body).

#### Ensuring all parts of the body were decontaminated

Participants’ actions were coded according to whether they did or did not miss any areas of their body; the number of times different parts of the body were missed was not counted. Results revealed that those in the guidance group missed fewer parts of their bodies than those in the no-guidance group (see Table 2). In the guidance group, no participants missed any parts of their body when going through the decontamination process, though some participants struggled to reach parts of their backs. By contrast, 9 of the 11 participants in the no-guidance group missed at least one part of their body. The most commonly missed body part was the hands (missed by 9 participants), with other missed body parts including the neck and right arm. A chi-square test revealed that the difference between groups was significant, (χ^2^ (1) = 22.84, p < 0.001).

**Table 2:** Number of participants who carried out the key steps of dry decontamination successfully in the guidance and no-guidance groups.

Group	Whole body decontaminated (n)	Top-down decontamination (n)	Sufficient blue roll used (n)
*Guidance*	10 (100%)	7 (70%)	10 (100%)
*No guidance*	2 (18%)	1 (9%)	0 (0%)

#### Decontaminating from the top down

It is important that the dry decontamination process is carried out from the head down, as this will help to ensure that areas of the body are not decontaminated more than once, thereby minimising the spread of a contaminant. Those in the guidance group were more successful at carrying out dry decontamination from the top down than those in the no-guidance group (Table 2). All participants in the guidance group worked from the top down, starting with their hands, then their face and neck, and then working down to arms, torso and back, and legs and feet. However, three participants went back over previously decontaminated areas of their body, with two participants going back over the hands when decontaminating the arms, and another participant going back over the face after decontaminating the neck, and over the neck after decontaminating the torso. Those in the no-guidance group also worked broadly top down; with 10 of the 11 participants starting with their face and neck. However, whilst participants initially started working from the top down, 10 of the 11 participants went back over at least one previously decontaminated area. A chi-square test revealed that the difference between groups was significant (χ^2^(1) = 9.27, p < 0.001).

#### Using sufficient blue roll

Participants in the no-guidance group used the same piece of blue roll to decontaminate more than one part of their body more often than did those in the guidance group (Table 2). Participants in the guidance group were instructed to use a new piece of blue roll for each part of their body (hands, face and neck, left arm, right arm, torso and back, left leg and foot, right leg and foot), but were not told how big a piece to use (e.g. one sheet or more). All participants followed the instructions to use a new piece of blue roll for each different part of their body, although they did occasionally go back over previously decontaminated areas, as noted above. By contrast, those in the no-guidance group did not use a new piece of blue roll for each part of their bodies. All of the participants in the no-guidance group used the same piece of blue roll on more than one body part at least once during the dry decontamination process. A chi-square test revealed that the difference between groups was significant (χ ^2^ (1) = 26.59, p < 0.001).

In support of the observation that those in the guidance group used more blue roll (and thus avoided cross-contamination of different areas of the body), the sheets of blue roll used in each session were counted, with participants in the guidance group (M = 12.6) using significantly more blue roll than participants in the no-guidance group (M = 8.27; t(16.4) = -2.82, p < 0.05).

#### Post-decontamination questionnaire

Initial analysis exploring the differences between male and female volunteers on questionnaire outcomes revealed that there were no significant differences in any of the items, with the exception of perceived acceptability of dry decontamination. Female participants reported higher perceived acceptability than males (t(16.74) = -2.63, p < 0.05).

**Fig 6:**
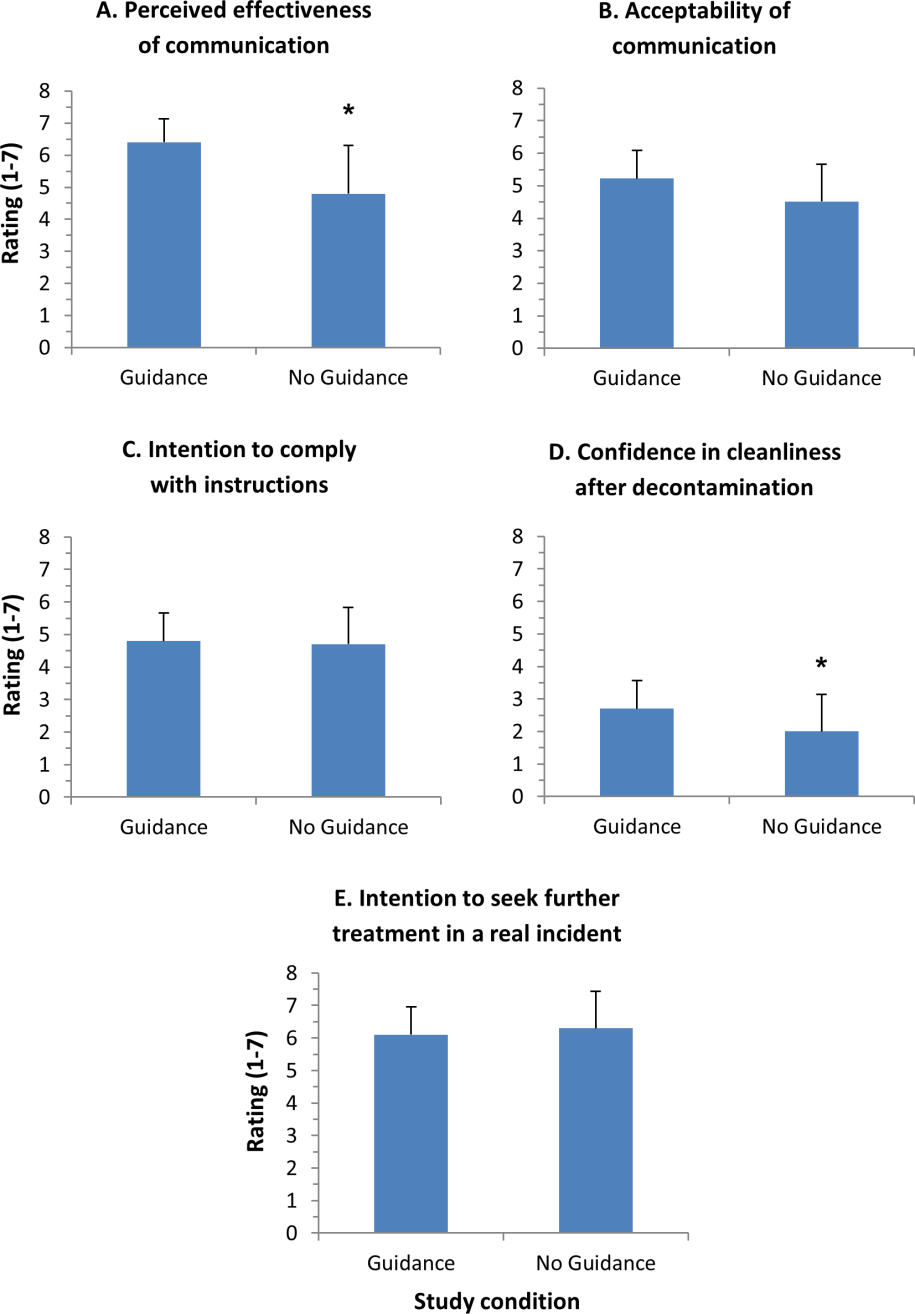
Post-decontamination questionnaire outcomes. Asterisks indicate significant differences between study groups (*p < 0.05).

Questionnaire outcomes by guidance group are presented in Fig 6. There were significant differences in the perceived effectiveness of communication (t(19) = -2.95, p < 0.05) and confidence in cleanliness (t(27) = -2.04, p = 0.05). For both questions, those in the guidance group reported higher ratings for communication effectiveness and cleanliness following decontamination when compared to the no-guidance group. There were no significant differences between the groups in perceived acceptability, intentions to comply, and intentions to seek further treatment (p > 0.05 for all). It is notable that scores were low for confidence in cleanliness, and high for intentions to seek further treatment in both groups (Fig 6D and 6E). These outcomes could have important implications for casualty management in real incidents, and this issue is returned to in the discussion.

## Discussion

*Study* 1 revealed that there was no significant difference in the effectiveness of dry decontamination between blue roll and incontinence pads. However, differences in the method used—blotting followed by rubbing resulting in the best performance when compared to matched controls—suggest that the key to performing effective improvised dry decontamination is to focus on optimising the method used; the type of absorbent material may be less important.

As the methyl salicylate spread differently on each participant’s skin, it was necessary to examine the effectiveness of each dry decontamination method when compared to a matched control on the opposite forearm of each volunteer. Results revealed that all experimental groups resulted in increased removal of methyl salicylate compared to the control groups. However, blotting and rubbing resulted in a significant reduction in the recovery of methyl salicylate in both swabs and tape-strips, when compared to control. This suggests that the combination method of blotting and rubbing was the most effective at removing the simulant contaminant. A companion study examining dry decontamination methods *in vitro* showed approximately similar removal percentages (≥70%) for a range of liquid chemicals [7]. To our knowledge the present study is the first of its kind to examine improvised dry decontamination methods in human volunteers.

Overall, simulant recovery was lower in all of the dry decontamination conditions when compared to matched controls, suggesting that dry decontamination, however performed, serves to reduce chemical exposure. However, Figs 4 and 5 also show that there were apparent differences in simulant recovery across the matched control conditions. These differences were only significant for the skin swab measure, where simulant recovery in the “rub” control condition was lower when compared to the “blot” and “blot and rub” conditions (p<0.05 for both). This suggests that overall; less MS was recovered from the skin in the “rub” condition than the “blot”, and “blot and rub” conditions. It is possible therefore, that the reduced availability of simulant on the skin of volunteers may account for the difference in simulant recovery between the different decontamination conditions. However, each decontamination condition was compared to a matched control condition, where an identical volume of MS was applied to the forearms of each volunteer at the same time. The behaviour of the simulant on the skin could then be affected by a number of factors, such as differences in topology and composition of the forearm skin surface, the density of forearm hair, small movements of the forearm by the volunteers (despite instructions to remain still) and environmental conditions on the study day. Whilst these factors may have introduced some variability in the behaviour of the simulant on the skin that could have influenced simulant recovery at the sampling sites, it is reasonable to assume that these factors would have acted equally on both the experimental and control arms for each participant. In future studies, carefully controlling for these potential confounders will add confidence to the outcome that a combination of both blotting and rubbing the skin resulted in the largest reduction in simulant recovery when compared to either blotting or rubbing alone.

In *Study 2*, those who received the dry decontamination guidance were able to carry out dry decontamination more effectively, by making sure all areas of the body were decontaminated, avoiding cross-contamination by working from the top down, and using sufficient blue roll. By promoting adherence to a safe and systematic decontamination protocol, the guidance would be likely to improve the effectiveness of the decontamination process. In addition, the provision of guidance resulted in an increased perception of having received sufficient information. This perception of having received sufficient information has been shown to contribute to increased willingness to comply with wet decontamination on the part of affected casualties [5,6], and could therefore contribute to increased willingness to comply with dry decontamination interventions.

Another factor that would affect the efficacy of the dry decontamination process during a real incident is whether those affected perceive dry decontamination as acceptable, and whether they would be willing to comply with the need to undergo dry decontamination. The results from the *Study 2* post-decontamination questionnaires revealed that those in the group receiving more guidance on the need and procedures for dry decontamination reported a higher perceived acceptability of dry decontamination as an intervention, and greater willingness to comply with the need for dry decontamination during a real incident, though the difference between groups was not significant. This is in line with previous research showing that the provision of effective communication and information during wet decontamination improves intentions to comply during a real incident [5,6].

There was also a significant difference between males and females in terms of the perceived acceptability of dry decontamination as an intervention, with females reporting significantly higher perceptions of acceptability than males. This is in line with research into compliance during a hypothetical chemical incident emergency, which showed that women were significantly more likely than men to accept the instructions of authorities and be compliant [15].

However, while the perceived acceptability of dry decontamination was generally high, confidence in cleanliness following dry decontamination was low in both groups. This suggests that, even though dry decontamination was perceived as an acceptable intervention, dry decontamination alone may not be enough to provide those affected with reassurance that they are clean following a chemical, biological, radiological or nuclear (CBRN) incident. This may lead them to seek further treatment; indeed, in the current study, intentions to seek further treatment following dry decontamination were also high. Research into real life incidents involving CBRN agents has shown that an increase in those seeking treatment for potential exposures can place significant additional demand on healthcare services, in some circumstances overwhelming hospitals and general practitioners’ surgeries [16]. It is therefore important that those affected feel they are clean, and do not feel the need to seek further treatment when they leave the scene of the incident. Further research should examine whether other factors, such as following dry decontamination with a full wet decontamination process, or providing those affected with increased information about the efficacy of the dry decontamination process, could reduce intentions to seek further treatment.

A limitation of this research is that there may have been a lack of ecological validity. Participants knew that it was a research study and it is therefore possible that they might react differently in a real scenario. For example, participants in the guidance group might have struggled to follow the instructions they were given had they received them during a real incident, when stress levels are likely to be higher. However, research into wet decontamination suggests that field exercises and field trials provide an effective way to test and develop communication plans [4,5,17,18], with such studies having good ecological validity. A second limitation of the present study is that the study group sizes were fairly small, when compared with some planning assumptions for mass casualty incidents. It is likely that an increased group size would only increase the need for effective guidance, as the ratio of responders to members of the public would be lower. Further, it is possible that interactions between group participants in each study condition could have influenced the outcomes in *Study 2*. Whilst no specific instructions were given to participants with regard to communicating with or following each other, observations of the trial suggested that participant interactions were minimal, and instead participants attempted to follow the instructions read aloud by the researcher. In real incidents involving large numbers of affected casualties and a much smaller numbers of responders, interactions between casualties may in fact be an asset to emergency responders who are attempting to lead dry decontamination processes. Casualties could be encouraged to assist each other in conducting the decontamination steps, and studies have shown that effective responder communication can promote such positive helping behaviours and adherence to decontamination protocols [5].

It is necessary to provide emergency responders and members of the public with guidance on how to carry out improvised dry decontamination effectively. These studies have shown that the provision of an absorbent material and some basic instructions is not enough; failure to provide effective instructions could lead to dry decontamination being carried out ineffectively, and may result in increased spread of a contaminant. Improvised dry decontamination is included as part of the UK Initial Operational Response Programme [19], and first responders will be instructed to use this as a default option prior to the commencement of any wet decontamination (unless a caustic or particulate agent is involved, or biological or radiological contamination is suspected). It is therefore essential that first responders be trained to recognise the importance of providing sufficient instructions to members of the public during dry decontamination, to ensure that dry decontamination is carried out effectively.

## Supporting information

S1 File Draft improvised dry decontamination guidance and instructions. (DOCX)

S2 File Study 2—Post-decontamination questionnaire. (DOCX)

S3 File Study 2—Scenario. (DOCX)
