# Supplementary material for: Volunteer trials of a novel improvised dry decontamination protocol for use during mass casualty incidents as part of the UK’S Initial Operational Response (IOR)
Source: PLoS One. 2017 Jun 16;12(6):e0179309. doi: 10.1371/journal.pone.0179309 (PMC5473560; doi:10.1371/journal.pone.0179309)
Supplement: S1 File — (DOCX) [file pone.0179309.s001.docx]

**Supplementary Data File 1**

**Draft improvised dry decontamination guidance and instructions**

**Performing improvised dry decontamination**

**Why is dry decontamination necessary?**

Research funded by the Department of Health has shown that disrobing as quickly as possible is the most effective way to remove a contaminant in a chemical incident. After disrobing, the use of a dry absorbent material to remove residual contamination from the skin is an effective step in the decontamination process. Using an absorbent material to carry out dry decontamination will ensure that as much of the contaminant as possible has been removed from casualties’ skin, and will help to prevent further spread of the contaminant to other people and places (e.g. acute hospitals).

**When is dry decontamination necessary?**

Dry decontamination should be considered the default process for an incident involving chemicals, unless casualties are showing signs of chemical burns or skin irritation, or have been contaminated with a particulate substance, rather than a liquid. Signs of chemical burns and skin irritation might include redness, itching or burning of the skin or eyes. In the case of chemical burns or contamination with a particulate, wet decontamination should be the default first option. The flow diagram in Figure 1 summarises the decision process that should be followed to select dry decontamination or wet decontamination.

**Decision tool to establish whether dry decontamination is appropriate**


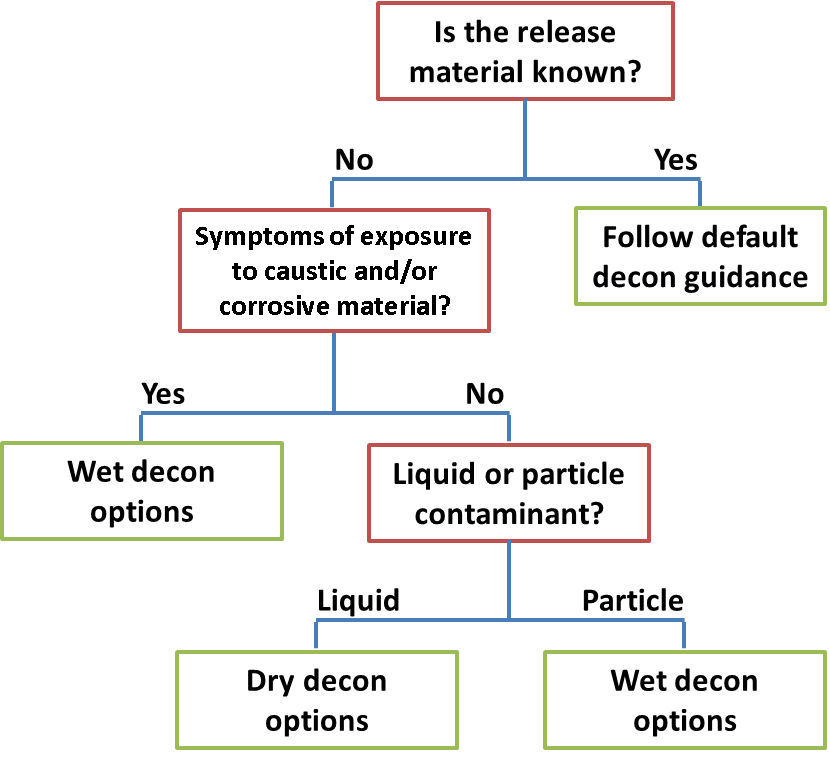


**IMPORTANT**

1. Stand up-wind of casualties when giving directions.

2. Sufficient blue roll should be used to avoid transferring contaminant from one part of the body to another.

3. Explain to casualties, “Please use this [blue roll] to remove the substance from your skin – we will now guide you through this process. Using [blue roll] to remove the substance from your skin will ensure that as much of the substance as possible is removed, which will prevent you suffering any adverse effects, and will also preventing spread of the substance to other people and places.”

**How should dry decontamination be carried out?**

Any absorbent material can be used to carry out dry decontamination. Blue roll has been shown to be effective for removing chemical simulants from the skin, and may be a good first option because it is readily available on ambulances and in emergency departments. However, any other absorbent materials (e.g. kitchen towel, towels, blankets) can also be used. If dry decontamination is necessary, guide casualties through the following steps:


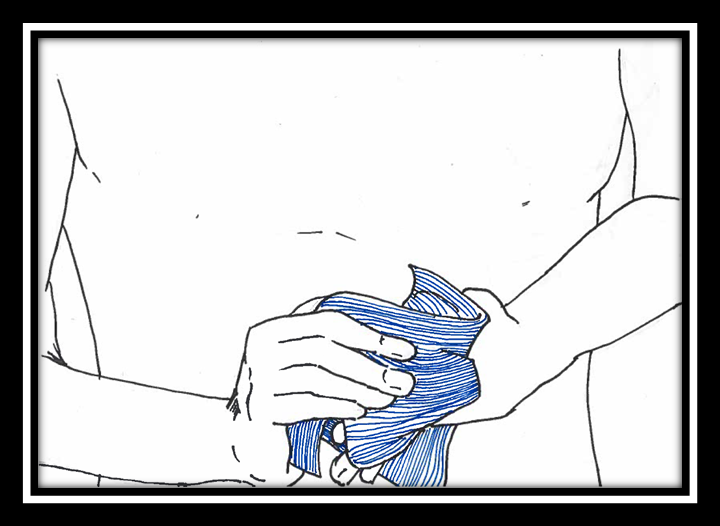

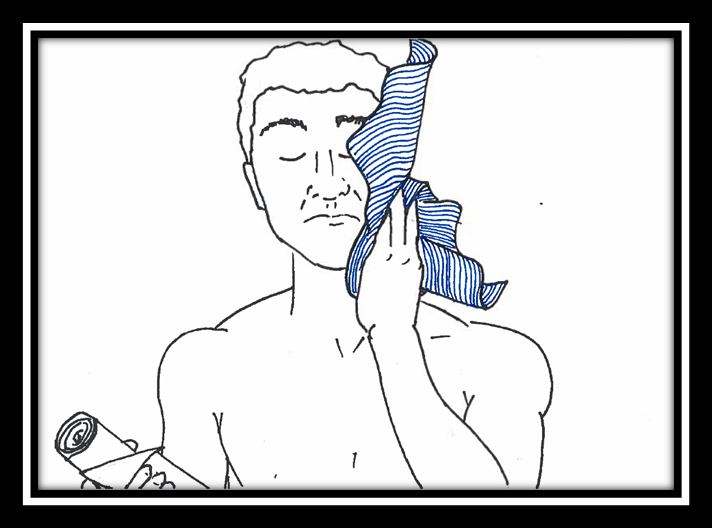

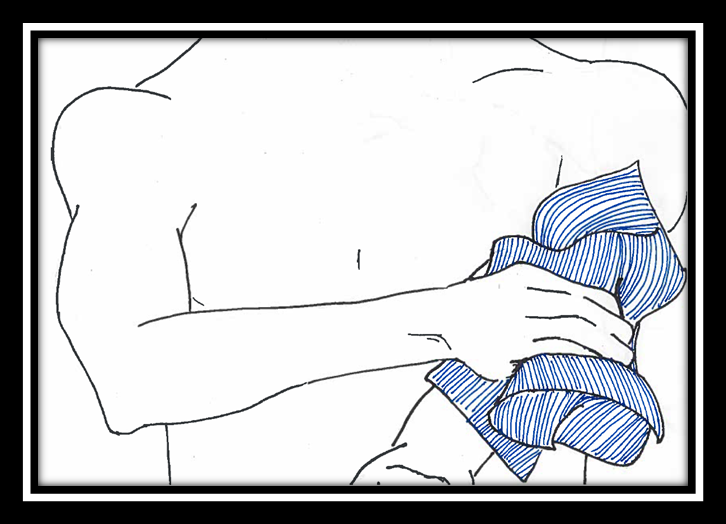

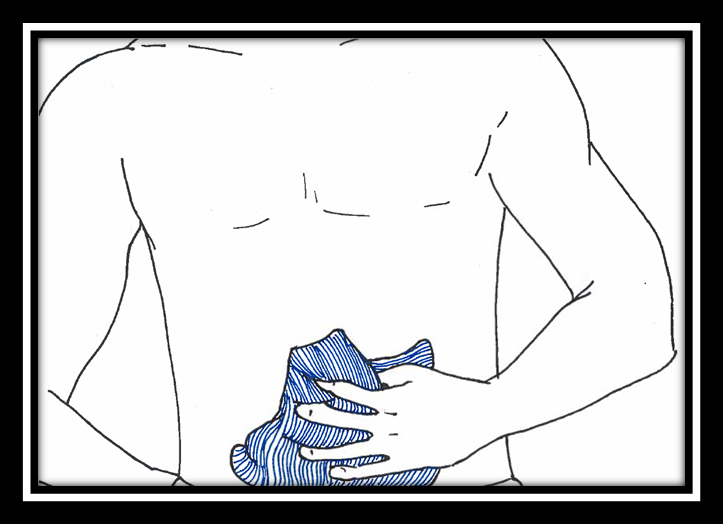

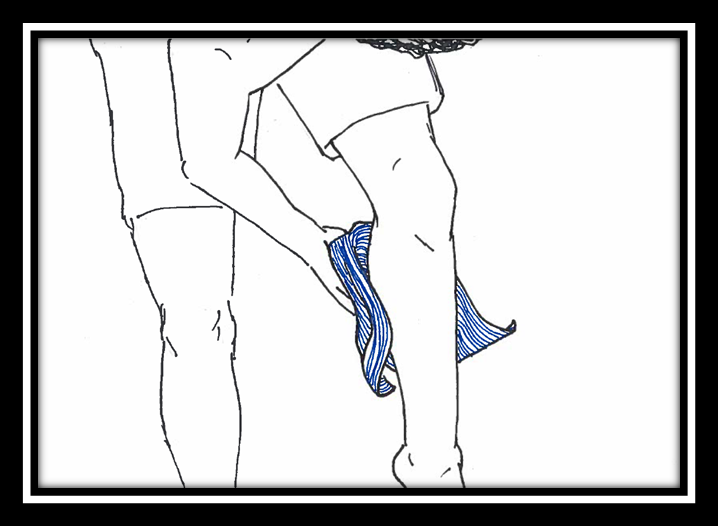


**1. Use some of the blue roll to blot and rub hands until**

**they are clean.**

**2. Use some more blue roll to blot and rub face and**

**neck until they are clean.**

**3. Use some more blue roll to blot and rub your left arm**

**until it is clean.**

**Now repeat for your right arm.**

**4. Use some more blue roll to blot and rub your torso**

**and back until they are clean.**

**5. Use some more blue roll to blot and rub your left leg**

**and foot until they are clean.**

**Now repeat for your right leg and foot.**
