# Supplementary material for: Volunteer trials of a novel improvised dry decontamination protocol for use during mass casualty incidents as part of the UK’S Initial Operational Response (IOR)
Source: PLoS One. 2017 Jun 16;12(6):e0179309. doi: 10.1371/journal.pone.0179309 (PMC5473560; doi:10.1371/journal.pone.0179309)
Supplement: S2 File — (DOCX) [file pone.0179309.s002.docx]

**Supplementary Data File 2**

**Study 2 - post-decontamination questionnaire**

**Participant experiences of dry decontamination**

Please read the following statements, and circle a number to show how strongly you agree or disagree with each one.

**1. Sufficient information was given about why dry decontamination was necessary**

| *Strongly disagree* | *1* | *2* | *3* | *4* | *5* | *6* | *7* | *Strongly agree* |
| --- | --- | --- | --- | --- | --- | --- | --- | --- |

**2. I understood why I was being asked to undergo dry decontamination**

| *Strongly disagree* | *1* | *2* | *3* | *4* | *5* | *6* | *7* | *Strongly agree* |
| --- | --- | --- | --- | --- | --- | --- | --- | --- |

**3. I was provided with sufficient information about how I was supposed to carry out dry decontamination.**

| *Strongly disagree* | *1* | *2* | *3* | *4* | *5* | *6* | *7* | *Strongly agree* |
| --- | --- | --- | --- | --- | --- | --- | --- | --- |

**4. I was clear about what I was supposed to do during dry decontamination**

| *Strongly disagree* | *1* | *2* | *3* | *4* | *5* | *6* | *7* | *Strongly agree* |
| --- | --- | --- | --- | --- | --- | --- | --- | --- |

**5. I felt comfortable using the blue roll to remove the simulated contaminant from my skin.**

| *Strongly disagree* | *1* | *2* | *3* | *4* | *5* | *6* | *7* | *Strongly agree* |
| --- | --- | --- | --- | --- | --- | --- | --- | --- |

**6. If this were a real incident, I would feel comfortable using the blue roll to remove the contaminant from my skin.**

| *Strongly disagree* | *1* | *2* | *3* | *4* | *5* | *6* | *7* | *Strongly agree* |
| --- | --- | --- | --- | --- | --- | --- | --- | --- |

**7. I felt embarrassed using the blue roll to remove the simulated contaminant from my skin.**

| *Strongly disagree* | *1* | *2* | *3* | *4* | *5* | *6* | *7* | *Strongly agree* |
| --- | --- | --- | --- | --- | --- | --- | --- | --- |

**8. If this were a real incident I would feel embarrassed using the blue roll to remove the contaminant from my skin.**

| *Strongly disagree* | *1* | *2* | *3* | *4* | *5* | *6* | *7* | *Strongly agree* |
| --- | --- | --- | --- | --- | --- | --- | --- | --- |

**9. I was able to quickly remove the simulated contaminant from my skin using the blue roll.**

| *Strongly disagree* | *1* | *2* | *3* | *4* | *5* | *6* | *7* | *Strongly agree* |
| --- | --- | --- | --- | --- | --- | --- | --- | --- |

**10. I found it easy to use the blue roll to remove the simulated contaminant from my skin.**

| *Strongly disagree* | *1* | *2* | *3* | *4* | *5* | *6* | *7* | *Strongly agree* |
| --- | --- | --- | --- | --- | --- | --- | --- | --- |

11. **I think that using blue roll is an effective way to remove the simulated contaminant from my skin.**

| *Strongly disagree* | *1* | *2* | *3* | *4* | *5* | *6* | *7* | *Strongly agree* |
| --- | --- | --- | --- | --- | --- | --- | --- | --- |

**12. If this were a real incident, I would feel confident that I was clean after using the blue roll to remove the contaminant from my skin.**

| *Strongly disagree* | *1* | *2* | *3* | *4* | *5* | *6* | *7* | *Strongly agree* |
| --- | --- | --- | --- | --- | --- | --- | --- | --- |

**13. I would be willing to undergo dry decontamination during a real life incident of this kind.**

| *Strongly disagree* | *1* | *2* | *3* | *4* | *5* | *6* | *7* | *Strongly agree* |
| --- | --- | --- | --- | --- | --- | --- | --- | --- |

**14. If this were a real incident, I would feel the need to seek further treatment after using the blue roll to remove the contaminant from my skin.**

| *Strongly disagree* | *1* | *2* | *3* | *4* | *5* | *6* | *7* | *Strongly agree* |
| --- | --- | --- | --- | --- | --- | --- | --- | --- |

**Thank you for taking the time to complete this questionnaire.**
