# Supplementary material for: Volunteer trials of a novel improvised dry decontamination protocol for use during mass casualty incidents as part of the UK’S Initial Operational Response (IOR)
Source: PLoS One. 2017 Jun 16;12(6):e0179309. doi: 10.1371/journal.pone.0179309 (PMC5473560; doi:10.1371/journal.pone.0179309)
Supplement: S3 File — (DOCX) [file pone.0179309.s003.docx]

**Supplementary Data File 3**

**Study 2 - scenario**

**Study 2 scenario**

***Condition 1 – no Instructions***

1. I want you to imagine you are waiting at a bus stop, for a bus to arrive.
2. Whilst you are waiting, you see a man carrying what looks like a back-pack type sprayer, with a hand-held nozzle.
3. The man approaches the bus stop and begins spraying you and those around you with an unknown substance, with an unusual smell.
4. The emergency services arrive within minutes and ask you to move away from the bus stop.
5. After asking you to remove the outer layer of your clothes, they provide you with blue roll, and ask you to use it to remove as much of the substance from your skin as you can.
6. We will now spray you with water to simulate a contaminant. Following this, please use the blue roll provided to remove the water from your skin, as you would if this were a real incident.
7. Please do not begin decontaminating yourself with the blue roll until I say ‘go’.

[**Spray**]

[If any questions are asked during the process:

“Please do the best you can with the resources provided.”]

***Condition 2 – Instructions***

1. I want you to imagine you are waiting at a bus stop, for a bus to arrive.
2. Whilst you are waiting, you see a man carrying what looks like a back-pack type sprayer, with a hand-held nozzle.
3. The man approaches the bus stop and begins spraying you and those around you with an unknown substance, with an unusual smell.
4. The emergency services arrive within minutes and ask you to move away from the bus stop.
5. After asking you to remove the outer layer of your clothes, they provide you with blue roll, and ask you to use it to remove as much of the substance from your skin as you can.
6. “Using the blue roll to remove the contaminant is an effective way to make sure that as much of the contaminant as possible is removed from your skin. This will help to reduce any adverse health effects from the contaminant, and will prevent spread of the contaminant to other people and places.”
7. We will now lightly spray you with water to simulate a contaminant. Following this, I will provide you with instructions on how to use the blue roll to carry out dry decontamination.
8. Please do not begin decontaminating yourself with the blue roll until I say ‘go’.

[**Spray**]

[Guide participants through the process using the decontamination instructions]
